# Synthesis and Morphology Control of Nanoporous Cu_2_O/Cu and Their Application as Electrode Materials for Capacitors

**DOI:** 10.3390/nano9030340

**Published:** 2019-03-02

**Authors:** Yuanwei Li, Xueyang Zhao, Hui Liu, Wei Li, Xiaojian Wang

**Affiliations:** 1 Institute of Advanced Wear & Corrosion Resistant and Functional Materials, Jinan University, Guangzhou 510632, China; jnulyw@stu2016.jnu.edu.cn (Y.L.); hc_sunboy@163.com (X.Z.); liuhuijnu@163.com (H.L.); 2 National Joint Engineering Center of High-performance Wear-resistant Metallic Materials, Guangzhou 510632, China

**Keywords:** nanoporous metals, dealloying, ligament size, copper

## Abstract

In this paper, nanoporous copper (NPC) was prepared by dealloying ZrCuAl metallic glass ribbons with HF acid solutions. The effect of dealloying time on the porous structures and thickness of the obtained NPC films was investigated. It was found that the ligament sizes of the NPC could be tuned in a range from 20 to 300 nm, and the thicknesses of the NPC films from 3.1 to 14.4 μm, with properly selected dealloying times. Furthermore, nanoporous composites made of NPC and nanoporous Cu_2_O were prepared by oxidizing the NPC with ethanol. The nanoporous composite electrodes exhibited superior charge-discharge performance and would have broad potential applications in flexible high-performance energy storage devices.

## 1. Introduction

Nanoporous metal is a unique type of porous material with pore sizes ranging from 0.1 to 100 nm. They exhibit a three-dimensional (3D) interconnected framework, large specific surface area, and excellent electrical conductivity [1], thus having wide applications in catalysis, sensing [2,3], actuating devices, surface-enhanced Raman scattering (SERS) [4], electrolysis, and supercapacitors [5]. Nanoporous metals can be prepared by templating method [6,7,8], electrochemical method [9], and dealloying method [10,11]. Among them, the dealloying method is a widely adopted method that selectively removing more active components from a precursor alloy. Precursor alloys include solid solution alloys and metallic glasses. According to the processing conditions, dealloying could be further classified into chemical dealloying [12], electrochemical dealloying [13], liquid metal dealloying [14,15,16], and vacuum extracting [17]. Using the chemical dealloying method, nanoporous metals such as nanoporous Au [18,19], Pt [20], Cu [21,22], Ni [23,24], Ag [25], and Co [26] could be prepared in a one-step chemical treatment.

Nanoporous metals, such as nanoporous gold (NPG), have proven to be a good supercapacitor current collector [27]. On the composite electrode made by depositing MnO_2_ onto NPG, the NPG effectively improved the conductivity compared to the MnO_2_ electrode. Moreover, the nanoporous structure of the composite electrode provided a high specific surface area, which facilitated the exchange of ions. The calculated specific capacitance 1145 F g^−1^ is very close to the ideal capacity of MnO_2_ of 1370 F g^−1^ [28]. Therefore, the capacitance of the transition metal oxide could be improved with the aid of nanoporous metals scaffolds. Driven by the reduction of manufacturing costs, less noble metals with good electrical conductivity, such as NPC or nanoporous nickel (NPNi), have received more attention lately. For examples, Zhao et al. prepared NPC by dealloying the Mg-Cu precursor alloy in HCl solutions. Wang et al. prepared NPC by electrochemical dealloying the Mg-Cu alloy in 0.2 mol L^−1^ (M) NaCl [29]. The ligament sizes of the obtained nanoporous structure range from 90 nm to 280 nm. Their results showed that dealloying conditions were very important for controlling the structure and composition of the NPC.

The transition metal oxides are promising pseudocapacitive materials because of their highly efficient redox charge transfer [30]. Xu et al. successfully prepared a Cu/CuO/Cu_2_O @nickel composite electrode by immersing a nickel foam in a CO(NH_2_)_2_ and Cu(NO_3_)_2_·3H_2_O mixed solution [31]. The obtained electrode showed an excellent performance with a maximum specific capacitance of 390.9 mF cm^−2^. In the current work, we synthesized a nanoporous Cu by dealloying ZrCuAl metallic glass ribbons in HF acid. The effect of dealloying time on the surface morphology and nanoporous structure were investigated. Nanoporous Cu_2_O/Cu composites were further prepared by oxidizing the NPC in ethanol. The nanoporous Cu matrix provided excellent electrical conductivity for the in situ formed Cu_2_O. Integrated as freestanding electrodes, the NP Cu_2_O/Cu ribbon exhibited excellent electrochemical performance for capacitors.

## 2. Materials and Methods

Zr_46_Cu_46_Al_8_ metallic glass ribbons were produced by single-roller melt spinning method. The well-prepared Zr (purity, 99.99%), Cu (purity, 99.99%) and Al (purity, 99.99%) were placed in an arc melting furnace (DHL-400, Shenyang, China), vacuumed to less than 5 × 10^−3^ Pa, and charged with high-purity Ar to a pressure of about 5 × 10^4^ Pa. The smelting was repeated 4 times to obtain a master alloy ingot with uniform composition. And the master alloy ingot was broken into alloy pieces and placed in a quartz tube with a nozzle at the bottom, placed in the middle of the induction coil of the quenching equipment (WK, Beijing, China), and the spray distance was adjusted. After vacuuming, a certain amount of high-purity N_2_ was charged to re-melt. When the melt reached the desired temperature, the gas pressure valve was opened and the melt was sprayed onto the surface of the rapidly rotating copper roll using the pressure difference between the top of the quartz tube and the cavity to obtain continuous metallic glass ribbons. The sizes of the ribbons were measured by a micrometer. The sizes of the ribbons were typically 30–40 μm in thickness and 3 mm in width. The amorphous structure of the prepared ribbons were confirmed by X-ray diffraction (XRD, UItima IV, Tokyo, Japan). The glass ribbons were dealloyed in a 0.2 M HF acid solution (AR, ≥40%, hengxing, Tianjin, China) under free corrosion conditions. Dealloying was continuously carried out in the HF solution at 70 °C and the dealloying time was normally less than 6 h. The dealloyed NPC were characterized by XRD (Cu Kα X-ray), scanning electron microscope (SEM, Phenom XL, Shanghai, China/ ZEISS ULTRA 55, Oberkochen, Germany) and transmission electron microscope (TEM, JEM 2100F, Tokyo, Japan) equipped with double spherical aberration correctors for both the probe-forming and image-forming objective lenses. The chemical compositions of the samples were analyzed using X-ray energy-dispersive spectroscopy (EDS, Phenom XL, Shanghai, China). The ligament sizes and porosity of the NPCs were determined based on the SEM images by image pro plus (IPP, MediaCybernetics, Rockville, MD, USA). At least 200 measurements were conducted for each sample.

The obtained NPC was oxidized in anhydrous ethanol at 40 °C for 24 h. Before oxidation, the NPC was cleaned with deionized water and absolute ethyl alcohol to remove the surface impurities and residual HF acid. The obtained NPCu_2_O/Cu were washed with deionized water 3 times and then dried at room temperature for 1 h for further characterizations.

The electrochemical measurements of the NPC/Cu_2_O electrodes were carried out on an electrochemical workstation (PRINCETON PARSTAT 4000 A electrochemistry workstation, San Diego, CA, USA) in a three-electrode electrochemical cell. The electrochemical measurements included charge and discharge, cyclic voltammetry (CV), and Electrochemical Impedance Spectroscopy (EIS). In the three-electrode system, a Pt sheet (16 cm^2^) was used as the counter electrode; the reference electrode was the saturated calomel KCl electrode (SCE, Rex-212, Shanghai, China), the working electrode was the sample, and the electrolyte is a 0.5 M Na_2_SO_4_ (AR, 99%, aladin, Shanghai, China) solution.

## 3. Results and Discussion

### 3.1. Chemical Dealloying

In the current work, Zr reacted with hexafluoros in HF acid and released hydrogen (Reaction 1), Al, and HF acid reacted to produce fluoaluminic acid and released hydrogen; the chemical reactions were as follows:

(1) $$6\mathrm{HF}+\mathrm{Zr}=H_{2}{\mathrm{ZrF}}_{6}+2H_{2}↑$$

(2) $$12\mathrm{HF}+2\mathrm{Al}=2H_{3}{\mathrm{AlF}}_{6}+3H_{2}↑$$

**Figure 1:**
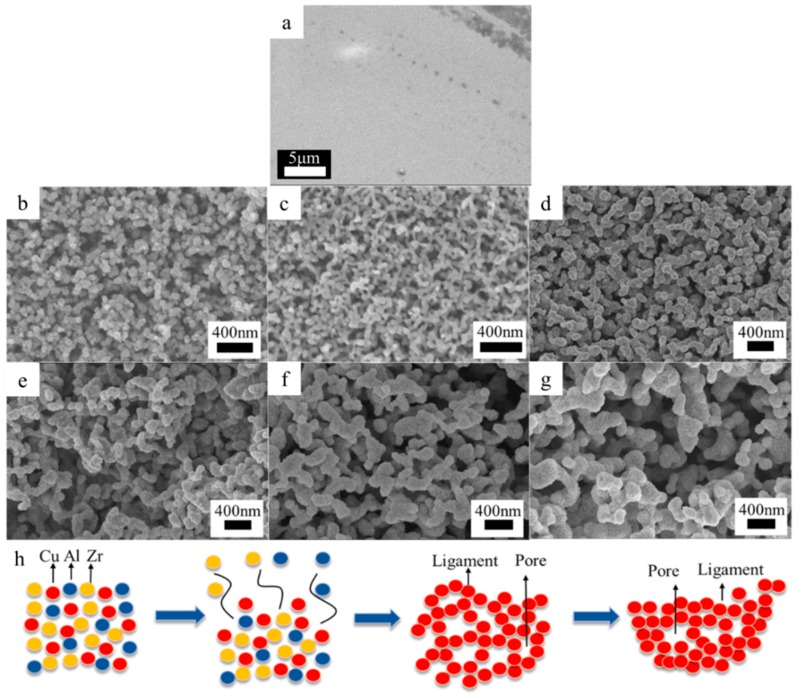
(**a**) The SEM image of the metallic glass ribbon; (**b**–**g**) Top-view of the NPC structures at different dealloying time (**a**) 5 min; (**b**) 10 min; (**c**) 40 min; (**d**) 2 h; (**e**) 4 h; (**f**) 6 h; (**h**) Schematic diagram of chemical dealloying mechanism.

Figure 1a is the surface morphology of the metallic glass ribbon before dealloying. Figure 1b–g shows the morphology of the NPC at different dealloying stages, from 5 min to 6 h. Significant coarsening had occurred for the extended periods of the dealloying process. In the beginning, such as dealloying for 5 min (Figure 1b), surface diffusion was driven by the reduction of surface energy. The dissolution of Al atoms and Zr atoms from the surface led to the accumulation of Cu atoms, thus forming copper-rich island structures. As the dealloying time increased, more Al atoms were dissolved, and the copper-rich islands began to form ligaments, and the structure evolved into a 3D network with a ligament size of 100 nm (Figure 1c). After 40 min of dealloying, the ligaments’ size significantly increased, as shown in Figure 1d. After 2h (Figure 1e) and 4h (Figure 1f), the pore sizes also significantly increased. The coarsening phenomenon can be explained as follows. With the aggregation of the Cu atoms, the area of Al and Zr atoms exposed to the HF solution became larger, and the dissolution of Zr and Al was accelerated. At this stage, more copper atoms were free on the surface of the sample and aggregate on the ligaments, and thus the ligament sizes became larger. Hence, the dissolution of the less noble metals and the coarsening of NPC ligaments occurred simultaneously and promoted each other. This phenomenon persisted after dealloying for 6h (Figure 1g). It is worth noting that the monolithic nanoporous layer was peeling off from the substrate, when the dealloying time was over 6 h.

Figure 1h illustrates the formation process of the nanoporous structure. At the first stage of dealloying, Al atoms and Zr atoms were dissolved into HF acid, and Cu atoms aggregate to form the ligaments and comprise the nanoporous structures. As the dealloying process continues, the pore sizes become larger, because of the dissolution of Zr and Al. With more vacancies appearing, Cu atoms continue to accumulate at the ligaments, and the ligaments’ size becomes larger, resulting in coarsening of the nanoporous structure.

**Figure 2:**
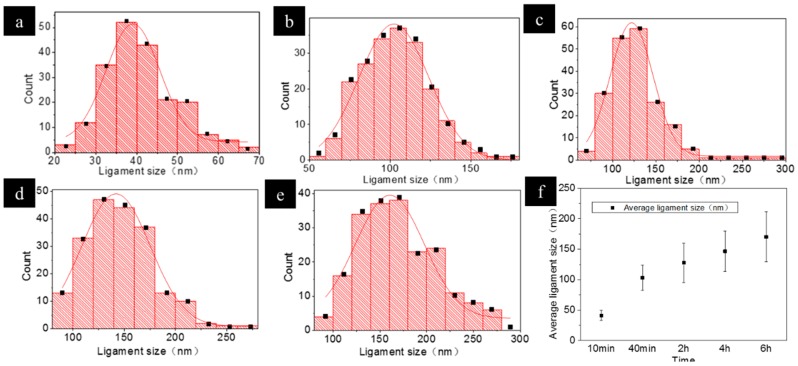
The distribution of ligament sizes of the NPC samples, dealloyed for 5 min to 6 h (**a**) 10 min; (**b**) 40 min; (**c**) 2 h; (**d**) 4 h; (**e**) 6 h; (**f**) Statistic of the average ligament sizes.

Figure 2a–e show the statistically estimated ligament sizes of NPC after dealloying for 10 min, 40 min, 2 h, 4 h, and 6 h, respectively. The average ligament sizes of the NPCs are 41, 103, 127, 146, and 170 nm, respectively. These results supported the observed dealloying process in Figure 1h. As shown in Figure 2f, the average ligament sizes of the NPC increased with the increasing dealloying time. The ligament sizes were found to follow a narrow Gaussian distribution. This result indicated that the obtained nanoporous structure was fairly uniform. After dealloying for 6 h, the ligament sizes increased to 140–190 nm, which is 4 times larger than the ligament sizes after dealloying for 10 min.

In order to understand the effect of dealloying time on the ligament sizes, the classic grain growth model was introduced to explain the relationship between the coarsening of ligament and the dealloying time. The coarsening process of the ligaments with dealloying time can be described with:

(3) $$d^{n}-d_{0}^{n}=kt,$$

where *d*_0_ is the initial ligament size, *d* is the measured ligament size, k is Boltzmann constant, *t* is the dealloying time, and *n* is the coarsening index. Since the initial ligament size is small, the initial ligament size can be ignored (*d*_0_ is 0). Based on the logarithmic form of Equation (3), the expression of *n* is:

(4) $$n=\frac{ln\left(kt\right)}{lnd},$$

The ligament coarsening index can be derived from Equation (4) based on the data in Figure 2. The coarsening indexes are shown in Table 1. 

It can be seen from Table 1 that the ligament coarsening indexes are almost a constant ~2.7. This result indicates that the ligament coarsening is a relatively stable process in dealloying. However, it is noted that the concentration of the dealloying solution was demonstrated to have a great influence on the ligament coarsening index. For example, in the AgMgCa system, the ligament coarsening index was up to ~11 in HCl solutions of a concentration less than 0.1 M [32]. When the concentration of HCl exceeded 0.1 M, the coarsening indexes decreased to ~2 and maintained a constant value. This result suggested that the coarsening of the ligaments mainly depend on the diffusion of the etching solution. 

**Figure 3:**
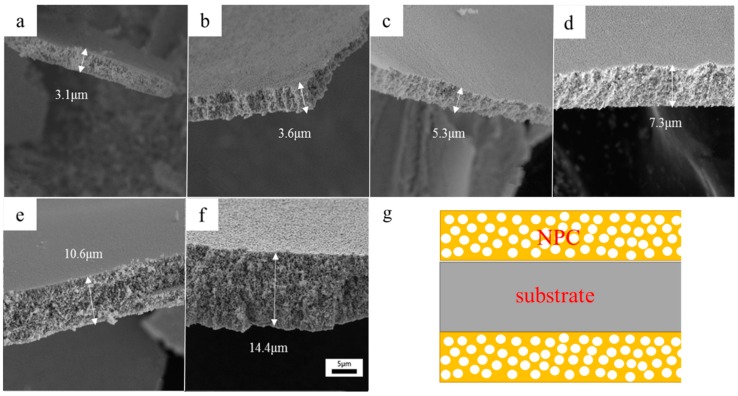
Cross-section images of the NPC at different dealloying time; (**a**) 5 min; (**b**) 10 min; (**c**) 40 min; (**d**) 2 h; (**e**) 4 h; (**f**) 6 h; (**g**) the structural schematic of sample cross-section during dealloying.

**Figure 4:**
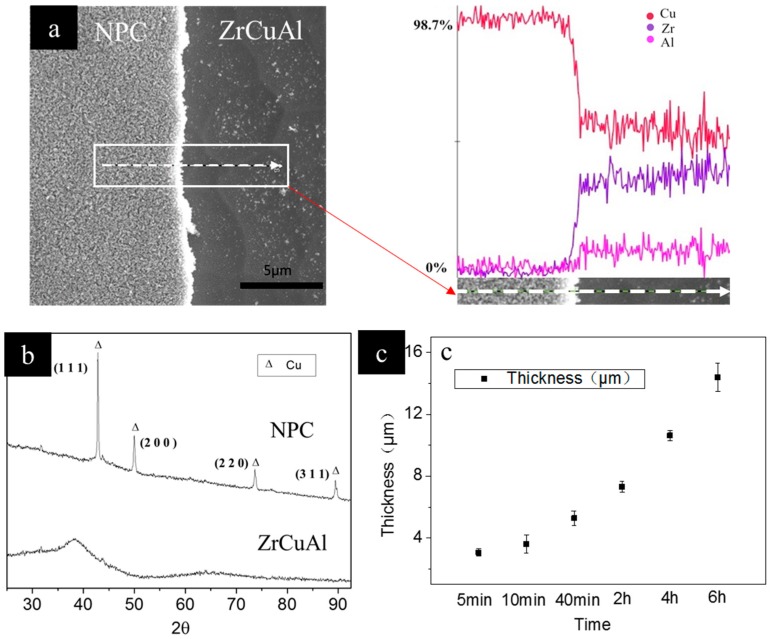
(**a**) The line scanning result of the NPC and the ZrCuAl, The subgraph is the line scanning path; (**b**) The XRD patterns of the NPC and the ZrCuAl; (**c**) The thickness variation of the NPC with dealloying time.

Figure 3a–f show the cross-section of the NPC after different periods of dealloying. The structural schematic of the cross-section of the ribbons after dealloying was shown in Figure 3g. It can be seen in Figure 3g that NPC is formed on both sides of the substrate and gradually grew into the interior, showing a sandwich structure. The cross-section of the NPC were examined after the NPC was scraped off the ribbon. It can be seen that the samples exhibited a regular 3D nanoporous structure. The formation of 3D nanoporous structure is due to the following two aspects: i), the active Al and Zr atoms dissolved into the HF solution forming vacancies, and then the vacancies coalescent due to the concentration gradient; ii) since the Al and Zr were homogenously distributed in the metallic glass ribbon, the vacancies formed pathways inside the sample, so that the etching solution continuously diffused into the sample and dissolved the Al and Zr inside. The thickness of NPC was measured at 10 positions of the dealloyed films. The average thickness values were also given. The thickness of NPC was only 3.1 μm after dealloying for 5 min. With further dealloying, the thickness of NPC increased to 14.4 μm after 6 h. Figure 4c shows the variation of the NPC thickness with increasing dealloying time. It can be seen that the rate of dealloying increased as the thickness of the NPC increased. This is mainly due to the continuous coarsening of the nanoporous structure, and due to that, the dissolution rate of Al atoms and Zr atoms was getting faster. This phenomenon would directly lead to the collapse of the nanoporous structure. The results indicate that the pore size and ligament size of porous metals can be controlled via dealloying time.

The EDS line scanning result of the ZrCuAl metallic glass ribbon after 6 h of dealloying is shown in Figure 4a. The EDS result shows that Zr and Al in the precursor are 46 at.% and 8 at.%, respectively. After dealloying for 6 h, the concentration of Zr and Al is reduced to zero, indicating that Zr and Al were completely dissolved into HF acid, and nanoporous copper was successfully obtained.

The XRD patterns of the ZrCuAl metallic glass ribbon and the NPC are given in Figure 4b. As can be seen from Figure 4b, both XRD patterns appear as small diffraction peaks after 30, 60 2θ and before 80 2θ. This phenomenon may be due to the fact that the slide and the adhesive are not clean enough during the experiment, which affects the XRD results. Before dealloying, the XRD pattern of the ZrCuAl metallic glass ribbon is a typical amorphous pattern with a diffusive scattering peak. After dealloying, the diffusive scattering peak of ZrCuAl metallic glass ribbons have completely disappeared, which means that the sample was crystallized. This is because the Zr and Al atoms in the amorphous ribbons were selectively removed, and the remaining Cu atoms devitrify. After dealloying, only the peaks of Cu could be detected from the XRD. The results of the XRD analysis are consistent with the EDS results. The XRD pattern indicates that the crystalline NPC is of a face-centered cubic (fcc) structure. The main diffraction peaks corresponding to the crystallographic planes are (111), (200), (220) and (311), respectively.

**Figure 5:**
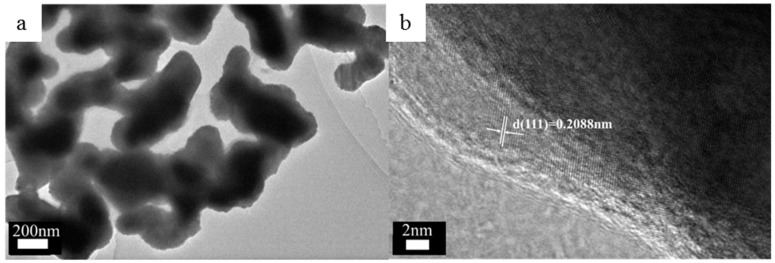
TEM images of the NPC after 6 h of chemical dealloying (**a**) porous structure of the NPC; (**b**) High resolution TEM image at the edge of NPC.

Figure 5a,b are the TEM images of the NPC. It can be seen that the NPCs show a uniform 3D bi-continuous nanoporous structure. The average ligament size was 228 nm, which is consistent with the result in Figure 1. The spacing of the lattice fringes was measured by the digital micrograph under HRTEM (Figure 5b). The latticespacing is 0.2088nm, which is equal to the plane spacing of the (111) plane in fcc Cu. These results also support the fabrication of NPC.

### 3.2. Electrochemical Performance of NPC/Cu_2_O

Transition metal oxides have proven to be good electrode materials because of their low cost and environmental friendliness [33]. However, transition metal oxides normally have low electrical conductivity. Therefore, improving the conductivity of transition metal oxides is the key to achieve high performance transition metal oxide electrodes [34]. Nanoporous metals have a large specific surface area and good electrical conductivity. The combination of transition metal oxides and nanoporous metals might improve the electrical conductivity of metal oxides. Next, the NPC that obtained after 4 h dealloying was oxidized to prepared Cu_2_O@Cu composite. Because the NPC obtained after 4 h dealloying has a certain thickness and larger porosity than the NPC obtained before, it is conducive to the oxidation reaction in anhydrous ethanol and the reaction between ions in the electrolyte and the electrode active substance.

**Figure 6:**
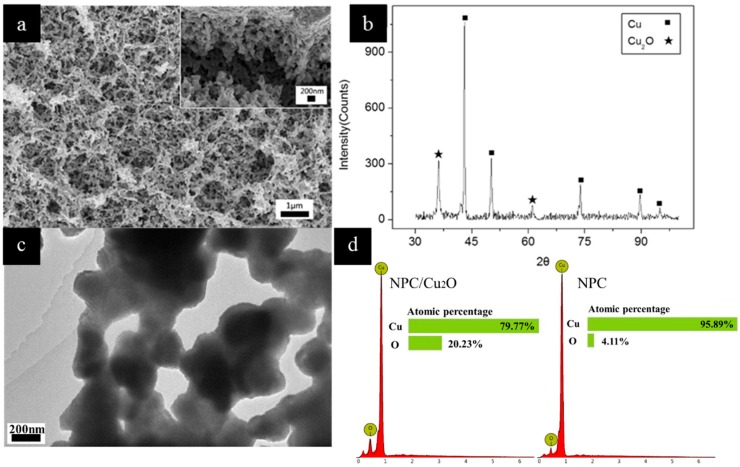
(**a**) the SEM image of the Cu_2_O/NPC, high magnification SEM image inserted; (**b**) The XRD pattern of Cu_2_O/NPC; (**c**) TEM image of the Cu_2_O/NPC; (**d**) The EDS results of Cu_2_O/NPC and NPC.

**Figure 7:**
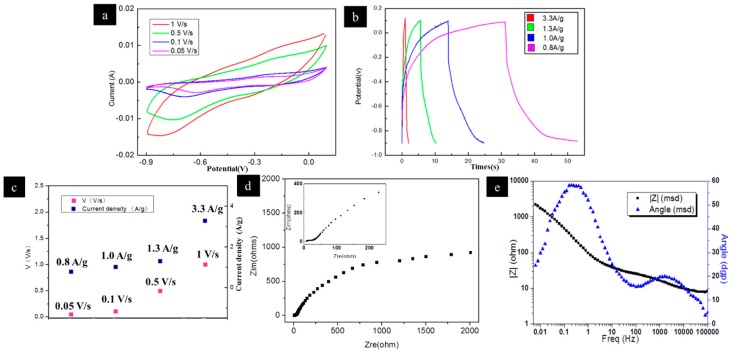
(**a**) CV curves of the NPC at different scanning rates in 0.5 M Na_2_SO_4_ solution; (**b**) Charge and discharge curves at different current densities; (**c**) CV scan rate curve and the trend of current density of charge and discharge; (**d**) EIS Nyquist plot of the NPC/Cu_2_O.The inner image shows more clearly region at high frequency; (**e**) EIS Bode phase plot of the NPC/Cu_2_O.

As shown in Figure 6a, the obtained composite maintained a 3D interconnected nanoporous structure with a high specific surface area. The XRD result (Figure 6b) showed that the NPCu_2_O had been successfully prepared. The TEM image (Figure 6c) of the composite also indicated its nanoporous structure. The EDS results (Figure 6d) showed that the oxygen content in the oxidized sample was significantly increased. The CV test results of NPC/Cu_2_O electrodes are shown in Figure 7a. The scanning speeds of the four curves were 1, 0.5, 0.1 and 0.05 V s^−1^, respectively. The active mass of the composite was 0.00027g. The specific capacitance was calculated with Equation (5).

(5) $$C=\frac{\int IdV}{2v\Delta Vm},$$

where *C* is the specific capacitance, *I* is the current, *V* is the voltage, *v* is the scanning speed, and *m* is the mass of the Cu_2_O. The calculation results show that the highest specific capacitance of the NPC/Cu_2_O composite is 190.93 F g^−1^. It can be seen from Figure 7a that the integral area of the CV curves increase with the scan rate. The main cause of this phenomenon is that the current increases as the scan rate increases. The increase of current is due to diffusion current (faradic current) and charge transfer current (non-faradaic current). As the scanning rate increases, the gradient of the CV curves also increase. This indicates that the resistance of the electrode is high and the electrochemical reaction of the composite produces faradic current. The calculated specific capacitance decreased as the scan rates increased. This phenomenon is mainly due to the fact that as the scanning rate increases, ions in the electrolyte can only reach the surface of the electrode and cannot enter the inside of the activities.

Figure 7b is of the charge-discharge curves of the Cu_2_O/NPC at different current densities from 0.8 A g^−1^, to 3.3 A g^−1^. The electrolyte was 0.5 M Na_2_SO_4_. It can be seen from Figure 7b that the charge and discharge speed increased with increasing current density. However, all of the charge and discharge curves were not standard. They were relatively asymmetric, where the charging time was longer than the discharging time. This phenomenon indicated that the Faraday reaction process was involved in the charge and discharge process. And the specific capacitance of the material was calculated by the following formula:

(6) $$C=\frac{It}{mV},$$

where *I* is the discharge current, *t* is the discharge time, *m* is the active mass, and *V* is the discharge voltage. Calculated by this formula, the calculated specific capacitance is 185.48 F g^−1^ when the current density is 0.8 A g^−1^, which is slightly lower than the maximum specific capacitance value obtained by the CV curve. So far, there have been many studies aiming at improving the actual specific capacitance of Cu_2_O. For instance, the graphene oxide/Cu_2_O/CuO nanocomposite was prepared and showed a specific capacitance of 173.4 F g^−1^ at a current density of 1 A g^−1^ [35]. The specific capacitance of the composite electrode obtained in this work is better than the graphene oxide /Cu_2_O/CuO composite. The TiO_2_ nanotube arrays (ED-TNAs) was used as the scaffold for Cu_2_O [36]. Cu_2_O nanoparticles were deposited on the ED-TNAs, which shows a specific capacitance up to 198.7 F g^−1^ at a current density of 0.2 A g^−1^. The specific capacitance of the NPC/Cu_2_O is similar to this composite electrode, but the preparation method is superior to the ED-TNAs /Cu_2_O, which is more convenient and environmentally friendly. These results suggest that the NPC/Cu_2_O composite electrode is comparable to other composite electrodes and provides an alternative electrode material for supercapacitors. At present, most electrochemical tests on copper oxides are around 0.5 V. In this study, all the electrochemical tests were performed in a wider potential range of 1 V, which indicated that NPC/Cu_2_O composite has high energy and power density. The energy density and power density are calculated by the following formula:

(7) $$E=\frac{1}{2}\times CU^{2},$$

(8) $$P=\frac{E}{t},$$

where *E* is the energy density, *C* is the specific capacitance, *U* is the test voltage, *P* is the power density and *t* is the discharge time. The calculated energy density is 26 Wh kg^−1^ and the power density is 1.3 W kg^−1^ at a current density of 0.8 A g^−1^. The EIS result of the NPC/Cu_2_O is shown in Figure 7d and (e). The EIS simulation was conducted according to the Nyquist plot in Figure 7e. The fitting results are shown in Table 2.

The ion transport process was analyzed based on the fitting results of Table 2. The diffusion of ions through the interface between the electrolyte and the active materials was represented by R_1_; the process of ions through the surface insulating layer inside the active material was represented by a parallel circuit of R_2_ and CPE_2_; the parallel circuit composed of R_3_ and CPE_3_ represented the diffusion of ions within the substrate. There is still a gap between the ideal specific capacitance of Cu_2_O (2247.6 F g^−1^) and the obtained NPCu_2_O in this work [31]. This may be due to the imperfect bonding between the obtained NPC/Cu_2_O and metallic glassy matrix after the chemical dealloying. Future research should focus on controlling the dealloying conditions and trying to obtain a well-bonded sample.

## 4. Conclusions

In this work, nanoporous copper (NPC) was successfully prepared by dealloying ZrCuAl metallic glass ribbons in HF acid. The coarsening of the ligaments was observed during the dealloying process. The dealloying time played an important role in the preparation of NPC, mainly affecting the morphology of the NPC and the stability of the porous structure. The ligament sizes of the NPC could be adjusted from 20 to 300 nm, and the thicknesses of the NPC film could be adjusted from 3.1 to 14.4 μm. The NPC was further oxidized in absolute ethanol to get the NPC/Cu_2_O composite. When the scanning speed was 0.005 V s^−1^, the specific capacitance of the NPC/Cu_2_O composite electrodes was 190.93 F g^−1^. The nanoporous composite electrode exhibited superior to electrochemical performance and has great potential as an application in flexible high-performance energy storage devices.

## Figures and Tables

**Table 1:** Ligament coarsening index at different time periods.

Time (min)	Ligament Coarsening Index
10	2.72
40	2.79
120	2.75
240	2.74
360	2.69

**Table 2:** EIS fitting results.

R_1_ (Ω)	CPE_2_ (Ω cm^−2^ S^−1^)	n	R_2_ (Ω)	CPE_3_(Ω cm^−2^ S^−1^)	n	R_3_	Chi Square
6.82	2.86 × 10^−3^	0.77	2592	6.57 × 10^−6^	0.54	24.79	1.36 × 10^−3^
